# Why do family firms dismiss their family CEOs? A perspective on kinship ties

**DOI:** 10.1371/journal.pone.0285029

**Published:** 2023-05-04

**Authors:** Xiaodong Yu, Shize Sun, Xirong Cheng, Yize Lin, Huan Li

**Affiliations:** 1 School of Business, Central University of Finance and Economics, Beijing, China; 2 School of Economics, Beijing Technology and Business University, Beijing, China; University of Guelph, CANADA

## Abstract

Existing studies have suggested that nonfamily CEOs are more likely to be fired from family firms, while we focus on why family CEOs are also fired from family firms. Using data from 455 listed Chinese family firms, we find that family CEOs with affinity ties are more likely to be dismissed as they are not genetically related to the family. The difference becomes greater when firm performance is poor or family ownership is high. These findings elaborate that business-owing family is not a group with aligned interests, that is, family members with different family identities are treated differently within family. Besides, existing studies have emphasized that the preservation of socioemotional wealth in family firms can affect firms’ operations, while this study further proposes that the preservation of socioemotional wealth can also have an impact on the business-owning families themselves.

## Introduction

Existing studies on family firms have concentrated on the differences between family and nonfamily CEOs [1]. Compared with family CEOs, nonfamily CEOs who don’t have family identity are less likely to be favored in family firms [2]. Therefore, when confronted with the circumstances of performance decline or conflicts with other managers, nonfamily CEOs are more likely to be dismissed by family firms [1]. With regard to this, extant studies from the perspective of socioemotional wealth theory have explained that for family firms aiming to promote family control and realize intergenerational transition, nonfamily members serving as CEOs will inevitably weaken the influence of the family on the firm [3]. Therefore, family firms have an instinctive rejection of "outsiders in power", leading to nonfamily CEOs are more likely to be fired by family firms [4].

However, many family firms have also dismissed family CEOs [5]. If nonfamily CEOs are more likely to be dismissed because they do not have a family identity, why would a family firm dismiss a family CEO? Extant studies typically emphasize that family firms have preference for family CEOs [4], but ignore that the family identities of family CEOs are not the same. Family CEOs who work in family firms have different family identities such as that of father, mother, son, daughter, spouse, siblings, and in-laws, among others. According to these differences in kinship types, such relationships can be divided into two categories: blood relationships linked by blood ties and affinity relationships linked by marital ties. To outsiders, even though family members have different family identities, they are all part of the family; however, inevitably, to family members, there are differences between family members who have different kinship ties with the family. Recent studies have focused on the role of genetic ties in family firm research, highlighting that the higher the degree of genetic relatedness, the more explicit and stronger their emotional closeness [6, 7]. Building on this logic, compared with blood relationships, affinity relationships based on marital ties do not have any genetic relatedness. Therefore, this study inquires whether family firms that distinguish between family and nonfamily CEOs also discriminate between family CEOs with blood relationships and those with affinity relationships.

To this end, this study examines whether family firms treat family CEOs with blood relationships differently from those with affinity relationships based on the perspectives of socioemotional wealth theory. We moved beyond the premise that a business-owning family is a group with aligned interests and explored whether family firms are inclined to fire CEOs served by affinity family members. On this basis, we further examined the influence of different business circumstances, such as family ownership and family firm performance, on the above question.

## Literature and hypotheses

### Socioemotional wealth preservation and family CEOs’ dismissal

Socioemotional wealth refers to the family firm’s noneconomic value that satisfy the emotional needs of family members [8]. According to Gómez-Mejía et al. [8], socioemotional wealth of family firms exists in various forms, including the family’s ability to exercise control and influence over the firm [9]; the satisfaction of family members’ sense of belonging and intimacy through working together within the firm [10]; and the ability to be altruistic toward family members and fulfill family obligations by providing family members with advantageous working opportunities and passing the family assets and traditions down to future generations [11, 12]. Socioemotional wealth theory proposes that while nonfamily firms focus on economic gains, the primary reference point for family firms is the preservation of socioemotional wealth [8]. For family members, their own control and identification of the firm and the emotional maintenance between members are of great importance; hence, they are willing to sacrifice economic benefits in exchange for noneconomic gains [13].

For family firms, an important way to preserve socioemotional wealth is to maintain family control over the firm [14]. Because family members tend to treat their firms as private property, they prefer to choose people they trust to take charge of daily operations in order to run their own firms well [15]. Therefore, family firms are more willing to place family members in CEO positions than nonfamily members [13]. The consistency between ownership and management endows the controlling family with greater discretion to exert the family’s will onto the firm’s strategic decision-makers to ensure that firm decisions conform to the pursuit of family-centered goals, such as the preservation of socioemotional wealth [2]. However, when nonfamily members serve as CEOs, they may be unaware of, or even ignore, the controlling family’s need to preserve socioemotional wealth and thus may implement strategies that are contradictory to this objective [4].

Based on the same consideration, family CEOs are unlikely to be fired once they have been placed on CEO positions. When a family firm underperforms, those with a family CEO attribute it to objective reasons, such as a downturn in the macroeconomic environment. In other words, the family umbrella protects family CEOs because of their familial ties. Conversely, nonfamily CEOs are more often held responsible for poor firm performance [1]. When a firm underperforms, nonfamily CEOs are more likely to be faced with the risk of being fired—at times as the scapegoat for controlling shareholders to stabilize the expectations of outside investors. Research has demonstrated that the tenure of family CEOs is three times that of nonfamily CEOs and that nonfamily CEOs are twice as likely to be fired [2].

### Genetic relatedness and kin selection

As mentioned above, family firms tend to place family members as CEO positions during operation [13]. Because of the consistent goals and interests, family members are inclined to have more trust among themselves, and hope that "one of their own" will take the important position such as CEO of the firm. At the same time, the altruistic complex among family members also makes them hope that family members can hold important positions in the firm, so as to obtain corresponding social status, economic returns and personal reputation [16]. Therefore, when the family CEO is dismissed, the emotional tendency to trust family members is deprived of the realization scenario in the family firm, and the need to care for and help family members cannot be met, which leads to the loss of socioemotional wealth [7]. Based on this, some studies have suggested that family firms are often reluctant to fire family CEOs [2].

However, family CEOs are not completely homogeneous, and family identities and status of family CEOs are not all the same. Researches based on evolutionary psychology interpret people’s emotional connection from the perspective of evolutionary theory and psychology, emphasizing that the close emotional relationship formed between family members is a psychological tendency of human beings in the process of evolution [6, 16–19]. This tendency is particularly prominent when there is a great genetic relatedness, that is, the existence of genetic relatedness means that individuals have stronger propinquity with each other. In other words, gene-based blood relationships provide natural emotional bonds for family members, enabling family members to maintain flesh-and-blood ties and strengthen emotional interactions, thus making emotional relationships based on genetic relatedness more stable and reliable [20], and arousing people’s stronger sense of trust in people with genetic relatedness [21, 22].

It is precisely because the connection of genes can bring more stable emotional relationships [23], there are inevitably differences between family CEOs with different identities. Compared with blood-related family CEOs, affinity family-member CEOs rely on the ties of marriage and are not genetically related to their families or family members. Hence, family firms’ intrinsic motivation and needs to favor family CEOs will inevitably favor blood family CEOs who have stronger genetic bonds. In other words, a high degree of genetic relatedness between blood relatives results in a stronger motivation toward altruism. As a result, the psychological cost of dismissing a blood-related family CEO is higher and more likely to result in a loss of socioemotional wealth. Based on this, we propose:

**Hypothesis (H1)**: Family CEOs with blood relationships are less likely to be fired than family CEOs with affinity relationships.

### Moderating effect of firm performance and family ownership

Existing studies based on kin selection theory suggest that, in the case of limited resources, people tend to allocate resources to relatives with close relationship and high degree of genetic relatedness [24]. At this time, the differences between family members with different relationships, such as blood relatives and affinity relatives will be more obvious. However, in the case of relatively abundant resources, more family members have access to family resources as resources are no longer scarce, making the above differences caused by genetic relatedness no longer significant [25]. It can be found that when the family resources are rich, business-owing family can treat the blood and affinity family members more equally [26, 27]. When resources are scarce, the limited resources are more concentrated in the hands of blood family members. At this point, affinity family members will face more intense competition for resources.

Extending this logic to family firms’ dismissal of family CEOs, when the family firm performs well, the resources owned by the family are relatively abundant. Under such circumstances, family firms can take into account both the blood-related and affinity family members at the same time. As a result, the family firms’ attitudes toward blood-related CEOs and affinity family-member CEOs are similar. When a family firm underperforms, however, family members have to be altruistic toward only blood relatives. In such circumstances, CEOs with affinity relationships are more likely to be dismissed. In other words, when a family firm underperforms, the differences between blood-related and affinity family-member CEOs become more significant. Based on this, we propose the following hypothesis:

**Hypothesis (H2)**: There will be a positive interaction between firm performance and the effect of blood-relatedness on CEO dismissal, i.e., blood-related CEOs will be even less likely to be dismissed (compared with affinity family-member CEOs) when performance drops.

As noted, there is a greater genetic relatedness between a blood family CEO and the business-owning family compared with an affinity family-member CEO; therefore, dismissing a blood family CEO would entail greater emotional loss. However, a family firm’s preference toward a blood family CEO might differ under different managerial contexts. When family ownership is low, the family has limited control and influence over a firm. Under such circumstances, although family members prefer a blood family CEO, with limited control, they are unable to fully implement family will or motivation over executive placement; hence, the controlling family’s inclination to retain blood family CEOs will become a lower priority. In contrast, when family ownership is high, family members have strong influence and control over the firm [28]. This not only enhances the voice of family members, but also makes it possible to exercise their biases within the family brought by kinship. Under such circumstances, the business-owning family is better able to exert the family’s will on strategic decision-makers; hence, they are better able to choose between blood-related and affinity family-member CEOs according to their need to preserve socioemotional wealth.

When family ownership is low, the business-owning family faces outside threats to the family’s control over the firm; hence, family firms are more motivated to place a family member in the CEO position to strengthen family control over the firm [7], though both blood-related and affinity family-member CEOs can help firms satisfy these needs. However, when family ownership is high, members are less concerned about maintaining family control. Under such circumstances, the business-owning family pays greater attention to the realization of family-centered goals, such as altruism toward family members [2]. Therefore, a business-owning family would prefer a blood family CEO who is genetically related to them; an affinity family-member CEO is less desirable. Based on this, we propose the following hypothesis:

**Hypothesis (H3)**: There will be a negative interaction between family ownership and the effect of blood-relatedness on CEO dismissal, i.e., blood-related CEOs will be even less likely to be dismissed (compared with affinity family-member CEOs) as family ownership increases.

## Methodology

### Sample

Our sample consists of publicly-listed family firms. We examined firms listed on the Small and Medium Enterprise Board, Growth Enterprise Board, and ChiNext Board of the Shenzhen Stock Exchange. The Chinese Stock Exchange consists of three parts: the Main Board, Small and Medium Enterprise Board, and Growth Enterprise Board. We use data from the Small and Medium Enterprise and Growth Enterprise boards because most firms listed on the Main Board are either state-owned or have strong government affiliation, making them unsuitable for this study [7, 29].

Because the definition and operationalization of family firms is often debated [30, 31], we used a multifaceted approach based on family ownership and involvement; that is, at least one additional family member works in the firm beyond the chairman, and the family owns at least 20% of firm shares [e.g., 32–34]. To ensure the accuracy of kinship and eliminate confounds, we omitted firms wherein the chairman was not a controlling member of the firm. The China Securities Regulatory Commission defines a family member as having a “controlling position” if that family member holds at least one of the four following positions in the firm: (a) director, (b) supervisor, (c) executive, and/or (d) key technical staff in the business. Based on these criteria, 682 family firms were initially identified. The Small & Medium Enterprise Board was established in 2004, so data were collected from 2004 onward. Since we focus on the topic of family firms dismissing family CEOs, we selected firms with family members as CEOs as the research sample. The final sample consists of 1942 observations of 455 families. The corporate governance and financial data involved in this study are from the China Stock Market & Accounting Research (CSMAR) database.

Among the 455 sample firms, 62.0 percent (282 firms) is listed on the Small and Medium Enterprise Board, and 38.0 percent (173 firms) is listed on the Growth Enterprise Board. Family firms from these two boards have similar family ownership level, age, performance, kinship structure, governance structures, and so forth. The firms on the Small and Medium Enterprise Board have an average of $370 million total assets, and those listed on the Growth Enterprise Board have an average of $160 million.

### Variable measurements

#### Dependent variable

*Dismissal of family CEO*. This variable is measured by examining whether family firms dismissed their family CEOs. If family firms dismissed the family CEO in a given year, “dismissal of family CEO” is marked as 1; otherwise, it is marked 0. It should be emphasized that being confronted with a situation such as performance pressure, even if the CEO offers to resign, it is likely that the resignation is submitted out of pressure from the firm [35]. In some cases, the firm claims that the resignation of the family CEO is caused by objective reasons, such as health problems, when, in fact, the family CEO is implicitly dismissed by the firm. To clarify the actual reasons for dismissal of CEO, this study observed how the CEO handled concurrent posts in the subsidiary firm and other concurrent posts in society. Usually, family CEOs of Chinese public firms simultaneously hold many posts in the firm’s subsidiaries or social entities. If the CEO resigns other positions, it is deemed factual that the CEO resigned because of health issues or other objective reasons. However, if the CEO does not resign from other concurrent posts after leaving a family firm, it is assumed that the main reason for the CEO’s resignation is not objective but rather the firm’s dissatisfaction with the CEO’s performance, which implies a situation in which the family firm voluntarily fired the CEO [36, 37].

#### Independent variable

*Family CEOs with blood relationships*. During the initial public offering (IPO), the China Securities Regulatory Commission requires that firms disclose the personal information of the chairman and the chairman’s relatives’ information and position in the firm in the prospectus. After listing, the firm must disclose the identity and background of all directors, supervisors, and executives who join or leave the firm each year in the resolutions of the board of directors, the resolutions of the board of supervisors, the annual report of the firm, and other public materials. Accordingly, we hand-collected data regarding the positions of the chairman’s relatives of each listed firm at the time of the IPO, updating them year-by-year by examining resolutions of the board of directors, resolutions of the board of supervisors, annual reports of the company, and other materials. Eight kinds of kinship relations are defined in this study, including parent, children, spouse, sibling, sibling-in-law, child-in-law, uncle/aunt–nephew/niece, and cousin relationships. Among them, parent, children, sibling, uncle(aunt)–nephew(niece), and cousin relationships are connected by blood ties with a high degree of genetic relatedness (i.e., blood relationships). Other relationships, such as spouse, sibling-in-law, and child-in-law are connected by marital ties and do not have genetic relatedness (i.e., affinity relationships). In addition, it should be emphasized that in Chinese families, only siblings of a biological parent can be called uncle/aunt. Thus, uncle/aunt here includes only siblings of the parent.

Family CEOs with blood kinship relations were measured through the kinship relationship between other family members who served in family firms as directors, supervisors, executives, and core technical personnel. Most family firms in China are first-generation family firms [7]; hence, determining the family relationships in firms is relatively obvious and simple. In the samples used in this study, 52 percent of family firms employ only one family member besides the chairman, 30 percent of family firms employ two family members besides the chairman, and less than 5 percent of family firms employ more than three members. In a relatively simple family structure, each family members’ suggestions and opinions will have a critical impact on the dismissal of a CEO; therefore, this study determined the kinship relationships between the family CEO and each family member. If the family CEO and at least one family member have a blood relationship, then the family CEO is deemed to have blood kinship relations, and the variable family CEO with blood kinship relations is marked as 1; otherwise, it is marked 0. As different family members can hold different positions in a firm, and may have different power and voice over the family firm, we also measured this variable through the kinship relationship between the family CEO and the chairman in a robustness test. For a given family firm in which the chairman is not concurrently the CEO, if there is a blood relationship between the family CEO and the chairman, family CEO with blood kinship is marked as 1; otherwise, it is marked 0.

#### Moderating variables

*Firm performance*. Consistent with previous research, we use return on assets (ROA) to measure firm performance [e.g., 38]. ROA is calculated as the ratio of net profit to total assets and is presented in decimal terms. Hence, the mean value of ROA is 0.05, indicating that the average level of firm performance is 5.00 percent. The standard deviation of firm performance is 0.05.

*Family ownership*. Consistent with existing research, we measure family ownership as the percentage of shares held by family members [e.g., 39]. When calculating total shares held by family members and total shares of the firm, we use data reported in the annual reports. The mean value of family ownership is 43.80, meaning that average family ownership is 43.80 percent and the standard deviation is 14.62.

### Control variables

We control for firm age and firm size (logarithmized) [e.g., 40] and for employee number [41]. We also control for financial leverage and firms’ capital adequacy [42]. On the governance side, we control for board size and independent director number. To control for family firms’ potential proximity to a succession event and intergenerational transition, we control for chairman age. Because executive compensation may affect the firm’s decision on the personnel departure and retention, we control for executive salary. In addition, we control for year (panel data from 2004 to 2017), industry [43], and area, as the institutional environment can strongly impact firms [44]. This information was omitted from the tables for parsimony.

## Results

### Descriptive statistics and correlation results

Prior to testing the hypothesis, this study first conducted descriptive statistics and correlation analysis on each variable, and the results are shown in Table 1.

**Table 1:** Descriptive statistic and correlations.

Variable	Mean	SD	1	2	3	4	5	6	7	8	9	10	11	12
1 Family CEO dismissal	0.06	0.24												
2 Blood CEO	0.69	0.46	-0.05											
3 ROA	0.05	0.05	-0.06	0.02										
4 Family ownership	43.80	14.62	-0.03	0.25	0.15									
5 Chairman age	53.08	8.68	-0.02	0.15	-0.01	-0.07								
6 Firm age	11.98	5.04	0.01	-0.03	-0.05	-0.07	0.19							
7 Employee number	2.96	11.02	-0.01	0.05	0.00	0.00	0.00	0.01						
8 Independent director number	3.05	0.41	-0.01	0.01	0.03	-0.04	0.01	-0.02	0.04					
9 Board size	8.19	1.31	-0.01	0.01	0.04	-0.14	0.07	-0.03	0.00	0.67				
10 Firm size	21.43	0.83	0.01	0.02	-0.06	-0.09	0.11	0.17	0.38	0.09	0.09			
11 Executive salary	12.15	0.56	0.06	-0.07	0.08	-0.04	0.06	0.23	0.24	-0.04	-0.06	0.50		
12 Debt/Asset rate	0.32	0.17	0.02	-0.03	-0.35	-0.10	-0.03	0.04	0.18	0.05	0.06	0.43	0.09	
13 Capital adequacy	0.27	0.80	0.00	-0.01	0.00	0.00	0.00	0.00	0.02	0.00	0.00	0.03	-0.02	0.02

**Note.** To facilitate the analysis, we take the natural logarithm of employee number, executive salary and firm size (total assets). Return on asset (ROA) and debt/asset rate are presented in decimal form. The mean value of ROA 0.05 stands for 5.00 percent. Family ownership is presented in percentage form. Correlations greater than 0.02 are significant at p < 0.05.

### Regression analyses

On the basis of correlation analysis, regression analysis is used to test the hypotheses. The results of the fixed-effects model testing the effects of kinship ties on family CEO dismissal are presented in Table 2. As shown in Model 1, there is a significant negative effect between family CEOs with blood relationships and family CEO dismissal (B = −0.064, *p* < 0.05), providing empirical support for Hypothesis 1.

**Table 2:** The effect of blood CEO on family CEO dismissal.

Variable	Family CEO Dismissal			
	Model 1	Model 2	Model 3	Model 4
Blood CEO	-0.064*	-0.089*	0.047	0.053
	(0.032)	(0.035)	(0.075)	(0.075)
Blood CEO*ROA		0.622^+^		0.790*
		(0.326)		(0.335)
Blood CEO*Family			-0.003^+^	-0.004*
ownership			(0.002)	(0.002)
ROA	-0.216	-0.673*	-0.227	-0.812**
	(0.163)	(0.290)	(0.163)	(0.296)
Family ownership	-0.001	-0.001	0.001	0.002
	(0.001)	(0.001)	(0.002)	(0.002)
Chairman age	-0.004*	-0.004*	-0.004*	-0.004*
	(0.002)	(0.002)	(0.002)	(0.002)
Firm age	0.018	0.017	0.018	0.018
	(0.012)	(0.012)	(0.012)	(0.012)
Employee number	-0.003	-0.003	-0.003	-0.003
	(0.003)	(0.003)	(0.003)	(0.003)
Independent director	-0.000	-0.002	-0.001	-0.003
number	(0.027)	(0.027)	(0.027)	(0.027)
Board size	0.005	0.006	0.005	0.007
	(0.010)	(0.010)	(0.010)	(0.010)
Firm size	-0.040^+^	-0.040^+^	-0.041^+^	-0.042^+^
	(0.023)	(0.023)	(0.023)	(0.023)
Executive salary	0.178***	0.176***	0.182***	0.179***
	(0.026)	(0.026)	(0.026)	(0.026)
Debt/Asset rate	0.078	0.078	0.080	0.082
	(0.071)	(0.071)	(0.071)	(0.071)
Capital adequacy	0.011	0.010	0.012	0.010
	(0.009)	(0.009)	(0.009)	(0.009)
Year	Control	Control	Control	Control
Industry	Control	Control	Control	Control
Area	Control	Control	Control	Control
Number of observation	1942	1942	1942	1942
Number of group	455	455	455	455

Note: + p < 0.1

* p < 0.05

** p < 0.01, and

*** p < 0.001, the values in parentheses are standard errors.

**Fig 1:**
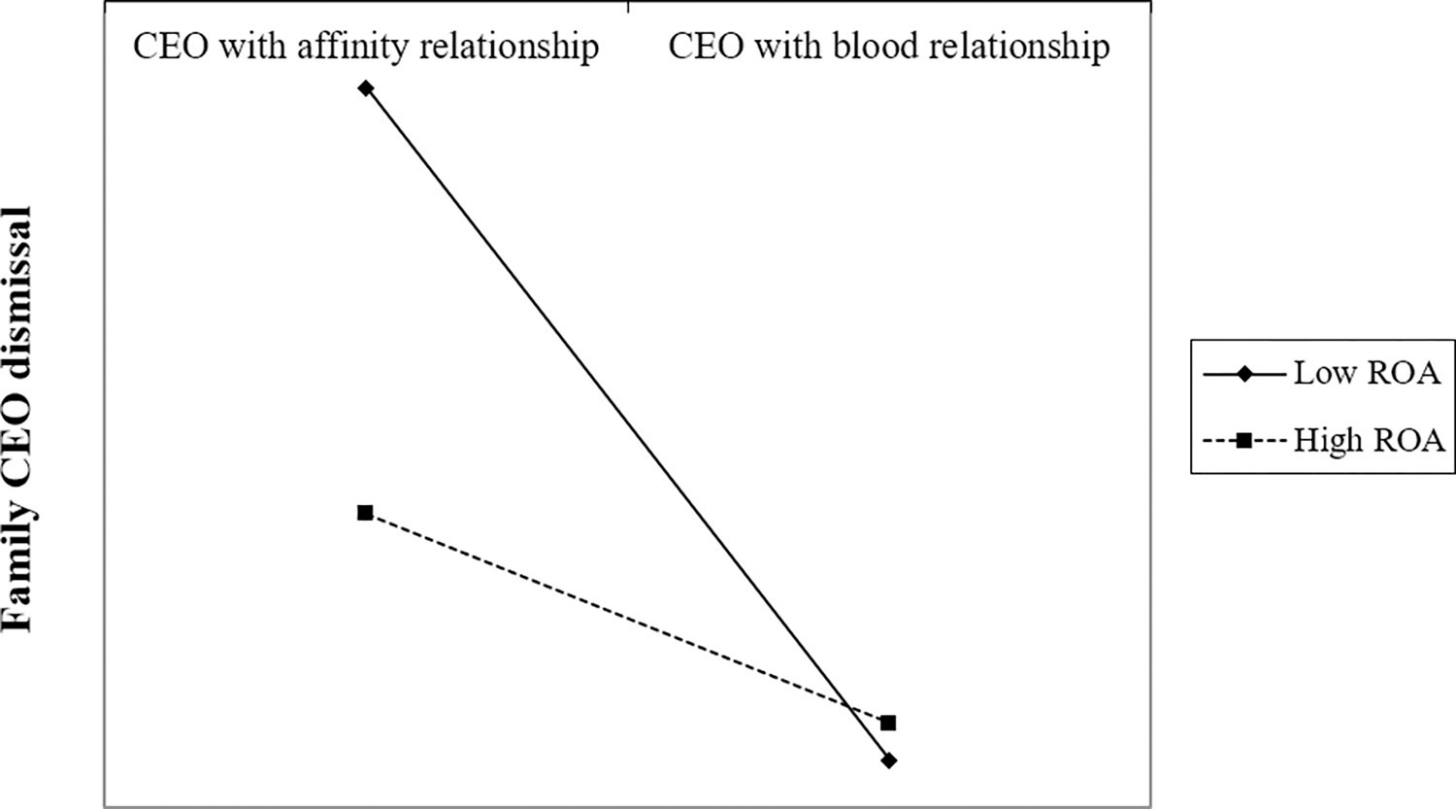
Moderating effect of return on assets (ROA) on family CEO dismissal.

Next, we tested the moderating effects of firm performance. Using the fixed-effects model, Model 2 of Table 2 indicates that the interaction of family CEOs with blood relationships and firm performance has a significant and positive effect on family CEO dismissal (B = 0.622, p < 0.1), supporting Hypothesis 2. We plotted both the interactions of family CEOs with blood relationships with moderators. The results of the interactions of blood CEO with moderators are presented in Fig 1 (t_high_ = -0.753, p_high_ = 0.452, t_low_ = −2.561, p_low_ = 0.011).

**Fig 2:**
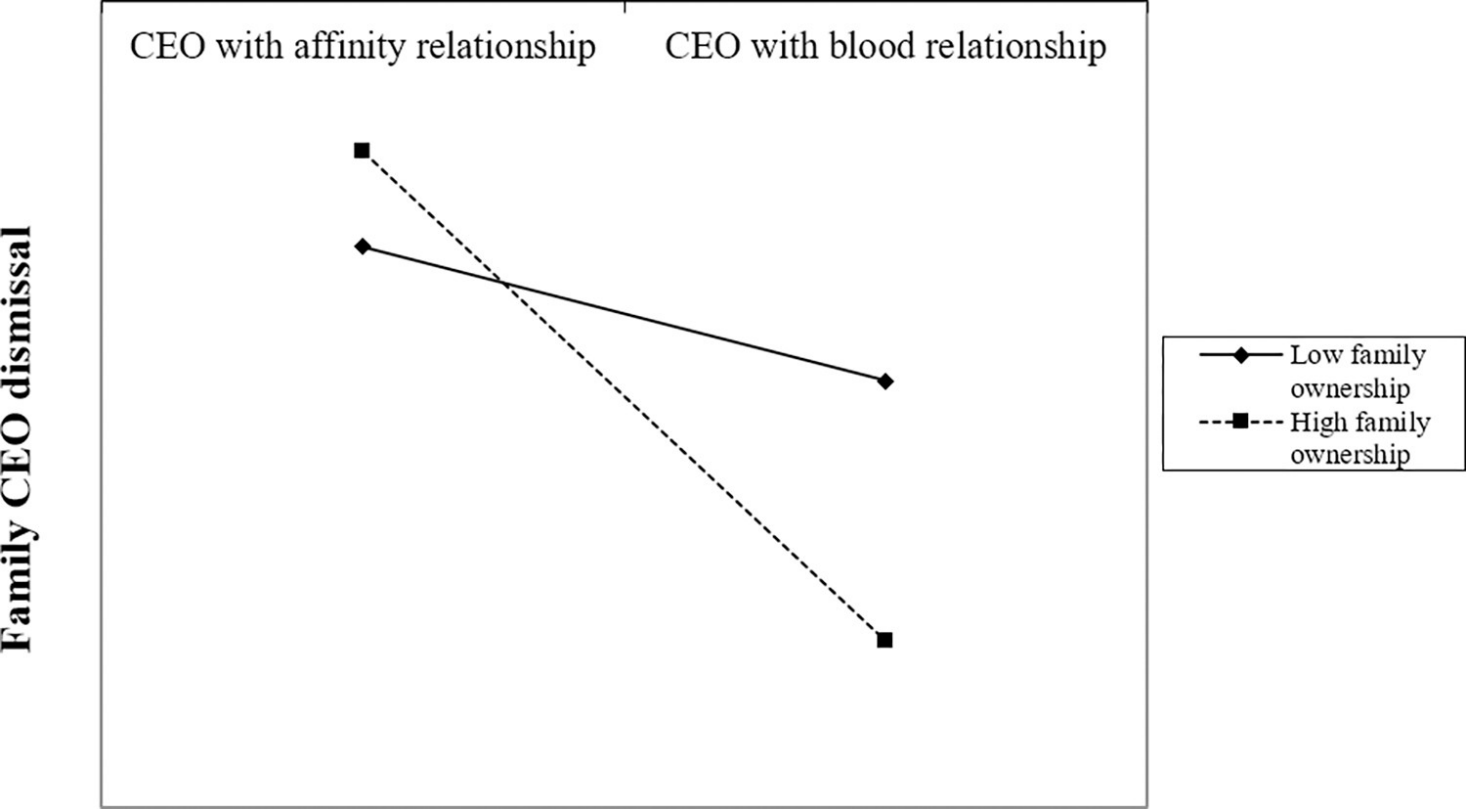
Moderating effect of family ownership on family CEO dismissal.

Turning to Hypothesis 3, Model 3 of Table 2 indicates that the interaction between family CEOs with blood relationships and family ownership has a significant and negative effect on family CEO dismissal (B = −0.003, p < 0.1), suggesting support for Hypothesis 3 (see also Fig 2, with the following simple slope significances: t_high_ = −2.566, p_high_ = 0.010, t_low_ = −0.745, p_low_ = 0.457).

In the full model, Model 4 of Table 2, the interaction between family CEOs with blood relationships and ROA presents a marginally positive, significant effect on family CEO dismissal (B = 0.790, p < 0.05), and the interaction between family CEOs with blood relationships and family ownership has a significant negative effect on family CEO dismissal (B = −0.004, p < 0.05), further providing marginal support for Hypothesis 2 and Hypothesis 3.

### Robustness tests

Several robustness tests were conducted. First, we repeated all analyses based on different family ownership to avoid biases introduced by our definition of family ownership set at 20 percent. We varied the family ownership threshold to higher than 15 percent and to 25 percent family ownership and higher, and conducted regression analysis using the research sample screened by the new definition. The regression results of 15 percent and 25 percent family ownership are shown in Model 1 and Model 2 of Table 3 respectively. Our findings were consistent across these operationalizations.

**Table 3:** Robustness tests.

Variable	Family CEO Dismissal			
	Model 1	Model 2	Model 3	Model 4
	15% ownership	25% ownership	Redefine blood CEO	Close/Distant relatives

Blood CEO/Close CEO	-0.071*	-0.066^+^	-0.536***	-0.079***
	(0.033)	(0.035)	(0.019)	(0.023)
Control variables	Control	Control	Control	Control
Year	Control	Control	Control	Control
Industry	Control	Control	Control	Control
Area	Control	Control	Control	Control
Number of observation	2104	1744	1942	1942
Number of group	469	434	455	455

Note: + p < 0.1

* p < 0.05

** p < 0.01, and

*** p < 0.001, the values in parentheses are standard errors.

We redefined “blood family CEO” by selecting various criteria. Considering that family members holding different positions in firms have differing levels of voice and power over firms, we also examined the relationship between family CEO and chairman, who is also the actual controller to judge whether the family CEO has a blood relationship. For family firms wherein the chairman is not also the CEO, if there is a blood relationship between the family CEO and the chairman, we regard the family CEO as a “blood family CEO” and the variable “family CEO with blood relationship” was coded as 1; otherwise, coded as 0. The results based on new criteria imply are shown in Model 3 of Table 3. These results show that there is still a significant negative relationship between blood family CEO and family CEO dismissal (B = −0.536 p < 0.001), meaning the results remain consistent across differing definitions.

We suggest that the reason family firms prefer CEOs with blood relationships is due to intimate connections between family members, which are based on care, altruism, and genetic relatedness. If this logic holds, then the closer the familial relationship, the less likely the family firms are to dismiss family CEOs. In Chinese families, in addition to dividing family members into blood relatives and affinity relatives according to whether there is blood relationship, family members are also divided into close relatives and distant relatives according to the distance of kinship. Thus, we tested whether family firms hold different attitudes between family CEOs with close kinship and those with distant kinships. If family CEOs with close kinship are also less likely to be dismissed relative to distant family CEOs, it would demonstrate that family firms indeed treat family CEOs differently based on blood ties. Close kinship was then identified by the existence of close relatives (i.e., parents, children, spouses, and siblings) holding positions as directors, supervisors, executives, and/or key technical staff in the family firm. In contrast, distant kinship was identified by relatives who are an uncle/aunt, nephew/niece, cousin, sibling-in-law, parent-in-law, or child-in-law, in addition to other family relationships. This classification of kinship is in line with Criminal Procedure Law of the People’s Republic of China, which defines husband, wife, mother, father, son, daughter, brother, and sister as “close kinship.” The results in Model 4 of Table 3 show that there is a significant negative relationship between family CEO of close kinships and family CEO dismissal (B = −0.079, p<0.001), indicating that the identity of family members and relatedness in genes indeed exert impact on family CEO dismissal.

In addition, this study also investigated whether, after a family CEO dismissal, the successor, if also a family member, is more likely to be a blood or affinity family member. If the proportion of blood successors is significantly higher than that of affinity successors after a family CEO dismissal, this also indicates that family firms treat family members differently based on kinship ties; that is, there is a tendency to transition perceptions of affinity family-member CEOs into blood family CEOs. The results supported this argument (χ^2^ = 44.45, p < 0.01).

## Discussion

The results of this study demonstrate that, compared to blood family CEOs, affinity family-member CEOs are more likely to be dismissed by family firms. These results indicate that, for family CEOs with blood relationships and affinity family-member CEOs, family firms typically tend to exhibit differentiated attitudes. In this regard, based on kin selection and socioemotional wealth perspectives, we proposed that the markers of intimate relationships, such as care and altruism among family members, are built on genetic relatedness. The more related the genes, the stronger and more obvious the close relationships between family members. Compared to affinity relatives, blood relatives have greater genetic relatedness, and thus have stronger intimate relationships. For family firms, having a blood family CEO could be a better way to preserve socioemotional wealth, whereas dismissing a blood family CEO induces higher socioemotional costs. Our findings provide evidence that the business-owning family is not an entity within which all members share completely consistent interests; instead, there remains an inherent difference between family members such that some family members are closer and the rest are more distant.

On this basis, the moderating effects of this study further demonstrated that family firms do not treat family members with different relationships equally. In the case of poor performance, the results showed a significant differential attitude of family firms toward family CEOs with blood and affinity kinship relationships. When bounded by limited resources, it is difficult for family firms to take care of all family members. In this case, family firms will give preference to members with blood ties, resulting in a significant difference in family firms’ treatment of family CEOs with blood and affinity kinship. When family ownership is high, the family has stronger control and influence over the firm, the family firm fully follows the family’s will regarding firm management and operations; the family’s motivation to differentiate between blood and affinity family-member CEOs will be carried out efficiently. In other words, in the case of higher family ownership, the difference between family CEOs with blood and affinity relationships was more significant, suggesting that family firms prefer family members with blood relationships.

Our study makes several contributions to the family firm literature. First, extant research tends to regard the business-owning family as a whole, whereas this study suggests that even within a family, members with marital identities are treated differently; that is, some family members are considered more “central” in the family, whereas others are considered less “central”. Compared with blood-related family CEOs, affinity family-member CEOs do not share genetic relatedness or blood ties with the family. Therefore, family firms who distinguish between family and nonfamily CEOs also distinguish between blood-related and affinity family-member CEOs. Second, this study suggests that the preservation of socioemotional wealth in family firms not only affects the operation of family firms, but also affects the business-owning families themselves. Existing studies have emphasized that the important goals of family firm operation are to maintain family control and extend family reputation, namely the preservation of socioemotional wealth. Therefore, family firms often choose a steady development strategy and pay attention to maintaining corporate image. However, this study provides further evidence that family firms’ preservation of socioemotional wealth also exerts an influence on the business-owning families. Compared with blood-related family CEOs, family firms suffer less socioemotional wealth loss when dismissing affinity family-member CEOs. Therefore, to preserve socioemotional wealth, family firms also discriminate family members with different identities within the family, and tend to choose family members with specific identities to join the business.

### Limitations and future research

This study has limitations, which also offer implications for future research. First, this study was conducted in a Chinese context, whereas future studies can attempt to replicate the results across multiple cultural contexts to verify the findings of this study. In particular, most family firms in China are first-generation family firms with relatively simple and clear kinship relationships. Thus, whether the findings of this study are applicable to family firms in other countries requires further analyses through future studies.

Second, this study explored the influence of family CEO identity on dismissal of family CEOs. Future research can focus on the influence of the succeeding CEO’s identity. Although our tests involved a test of the successor’s identity, many questions remain unanswered. For example, after dismissing the family CEO, why do some family firms appoint another family member to succeed, and why do some prefer outsiders? When a nonfamily member becomes the CEO, what kinds of adjustment and changes occur within a family firm?

Third, our sample included only listed family firms. Future studies could examine data of unlisted family firms for verification and comparison. Compared with listed family firms, unlisted family firms receive less regulatory attention from government and external shareholders. Will the influence of kinship relations be more obvious for unlisted family firms? All these issues need to be further explored in future research.

## Supporting information

S1 File (DTA)
